# Pilot study on increased adherence to physical activity on prescription (PAP) through mindfulness: study protocol

**DOI:** 10.1186/s13063-018-2932-9

**Published:** 2018-10-17

**Authors:** Peter Nymberg, Eva Ekvall Hansson, Emelie Stenman, Susanna Calling, Kristina Sundquist, Jan Sundquist, Bengt Zöller

**Affiliations:** 1 Lund University/Region Skåne, Centre for Primary Health Care Research, Jan Waldenströmsgata 35, Skåne University Hospital Malmö, University Hospital, SE-205 02 Malmö, Sweden; 2 Department of Health Sciences, Division of Physiotherapy, Lund University, Baravägen 3, SE-221 00 Lund, Sweden

**Keywords:** Physical activity, Motivation, Health care, Lifestyle change

## Abstract

**Background:**

In the Swedish population aged between 50 and 64 years only 7.1% reach the recommended level of physical activity. Physical activity on prescription (PAP) has been used in Sweden since the beginning of the twenty-first century with moderate adherence of approximately 50%. Mindfulness seems to affect motivation to and satisfaction with physical activity. The aim is to test the feasibility of a study in routine care; i.e. to test if mindfulness can improve adherence to PAP, measured by changes in physical activity.

**Methods/design:**

We will include 90 sedentary individuals, aged 40–65 years, from primary health care centres in Sweden. Individuals will be randomised to only PAP, mindfulness and PAP or mindfulness only. The PAP group will be based on patients’ preferences. The mindfulness groups will meet once a week for 8 weeks and practise 20 min of individual training per day. There will not be any motivational interview or physical activity on prescription in the group assigned to only mindfulness.

The participants will complete the Five Facet Mindfulness Questionnaire, the Insomnia Severity Index and also answer questions concerning their lifestyle. Physical activity will be measured by ACTi Graph GT1X activity monitor at baseline and after 3 and 6 months. Patients with a severe psychological disease, unstable angina or a recent myocardial infarction will be excluded. The main outcome will be adherence to PAP in an ordinary primary health care setting. In this pilot study, we will also evaluate measures such as the recruitment rate, number of dropouts and adherence to mindfulness practice.

**Discussion:**

This study is the first to explore the effect of mindfulness on adherence to PAP and test the feasibility of the study design.

**Trial registration:**

ClinicalTrials.gov, NCT02869854. Registered on 26 August 2016.

**Electronic supplementary material:**

The online version of this article (10.1186/s13063-018-2932-9) contains supplementary material, which is available to authorized users.

## Background

A lifestyle with an adequate amount of physical activity can decrease the risk of cardiovascular illness [1, 2] and improve perceived quality of life [3]. Although people in northern Europe are physically active [4], they report more sedentary time than their southern European counterparts [5]. A study concerning sedentary behaviour among 50- to 64-year-old Swedes showed that only 7.1% of the 948 participants fulfilled the World Health Organization (WHO) recommendations for physical activity [6]. In Sweden, the health care service recommends the use of physical activity on prescription (PAP) as a complementary treatment to motivate patients to increase their activity level; the treatment addresses both primary and secondary prevention of sickness. The written prescription can be a proposal for an activity or an extensive solution with a supportive structure, depending on the patient’s needs and level of motivation. PAP is associated with up to a 60% increase in activity levels, but, unfortunately, the increase is not sustainable over time [7, 8]. A systematic review estimated that one must treat 12 sedentary adults with a physical activity promotion intervention in order to make one of them achieve the recommended physical activity level at 1 year follow-up [9]. In addition, PAP seems to be most effective in individuals who are already slightly active. Satisfaction plays a crucial role in changing a behaviour; this has been observed in smoking cessation [10], weight loss [11] and physical activity [12]. Satisfaction is increased both by the awareness in a specific positive situation and by the reduction of negative thoughts, e.g. about physical activity [13]. To experience increased satisfaction with physical activity, it seems necessary to be aware of the present; something which may be facilitated by practising mindfulness [14]. All people have varying capacities to attend to and to be aware of the present moment, which is called dispositional mindfulness, and it is an intrinsic but a modifiable trait [15]. Mindfulness can be exerted as sitting meditation but also as an approach to everyday life [16]. The practice of mindfulness can increase recognition of mental experiences in the present moment by self-regulation of attention—meaning that it is preserved on immediate experience. It might also enable individual traits such as increased curiosity, openness and acceptance because of the orientation toward one’s experiences in the present moment [17]. Being mindful in a specific situation and satisfaction are suggested to be consecutive mediators for the path between possessing a dispositional tendency to be mindful and physical activity [14]. This might explain why self-reported mindfulness seems to mediate the relationship between intrinsic motivation and the physical activity level [18]. In other words, practising mindfulness can make it easier to experience satisfaction with physical activity and, in this way, support the change from a physically inactive behaviour to a physically active one. Conversely, trials have shown that regular exercise can also lead to increased dispositional mindfulness [19]. Thus, mindfulness may have a crucial role in motivation by reinforcing satisfaction with physical activity. However, the matter of causality is still unclear.

## Methods/design

### Aim

The aim of this pilot study is to test the feasibility of the main study in regular care. The overall aim of the main study is to examine whether a mindfulness programme can increase the adherence to PAP in an ordinary primary health care setting. To achieve this, we will compare three different intervention groups: PAP, mindfulness and a combination of PAP and mindfulness. A secondary aim is to evaluate differences between the three intervention groups regarding changes in health-related parameters related to physical activity, such as blood pressure, weight, lipid profile, biomarkers, gene and protein expression, self-rated health, insomnia and mindfulness.

### Setting

The pilot study involves three primary health care centres in the county of Skåne (Scania) in southern Sweden. In total, there are approximately 164 primary health care centres in the county. Scania has approximately 1.2 million residents, 410,000 of whom are aged between 40 to 65 years old.

### Participants

The main inclusion criterion for participants is that they are insufficiently physically active individuals aged between 40 to 65 years. The criteria of physical activity are defined according to the WHO guidelines regarding physical activity recommendations, where the lower limits are set to < 150 min per week of moderate intensity or < 75 min per week of high intensity.

Exclusion criteria are dementia, serious mental disorder, newly diagnosed untreated unstable angina pectoris or myocardial infarction within 6 weeks prior to study entry. Individuals with a physical disability which decreases their ability to perform physical activity will be excluded as well as individuals who cannot master the Swedish language in speech and writing.

### Interventions

Intervention A comprises PAP only, which is treatment as usual in physically inactive patients [7, 8].

Intervention B comprises PAP and mindfulness. This involves treatment as usual with the addition of a 2-h-long mindfulness group session once a week for 8 weeks, as well as 20 min of daily practice. The mindfulness course is based on both mindfulness-based stress reduction (MBSR) and mindfulness-based cognitive therapy (MBCT) and includes meditative exercises. The patients will receive instructions concerning the daily practice with meditative exercises in the web-based programme. The instructions include breathing technique and body scan [20].

Intervention C comprises the same mindfulness course as intervention B, but without PAP.

**Fig. 1:**
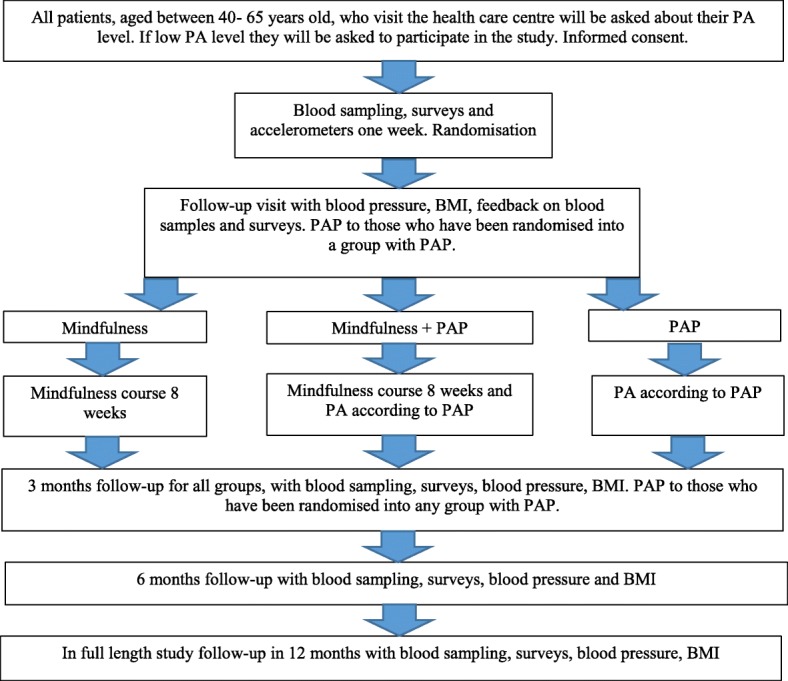
Study design

A flowchart describing the study design is presented in Fig. 1.

The randomisation will be stratified by the patients’ age and sex, and there will be three age groups: 40–49, 50–59 and 60–65 years. The randomisation will be done using the minimisation method with a random element [21] and the statistical program STATA version 14.1.

### Procedure

Placards will be placed in the participating primary care centres to raise interest among the patients to participate in the study. All patients within the 40–65 age group, regardless of the reason for their visit, will be asked about their physical activity level. Patients who regard themselves as insufficiently physically active (according to WHO’s recommendation) will be asked to participate in the study. Eligible patients will be contacted by a nurse to be informed about the study and give their written consent if they wish to participate further. Patients who are willing to participate will be asked to come for fasting blood sampling and will receive accelerometers, which will be attached to their hip during the next 7 days apart from when bathing and sleeping at night. Mindfulness will be measured by the Five Facet Mindfulness Questionnaire (FFMQ) [22], and insomnia will be measured with the Insomnia Severity Index (ISI) [23]. The survey about lifestyle habits includes short questions about alcohol, food, physical activity and eating habits. All the surveys will be in Swedish. The patients will fill out the questionnaires during the period prior to their follow-up visit after approximately a week. During the time between blood sampling and the follow-up visit, patients will be randomised to one of the three intervention groups. The patients will be informed about the randomisation at their follow-up visit. At this visit, the blood pressure (in sitting position after 5 minutes, in the right arm) and body mass index (BMI) will be measured. The patients will also get feedback on the fasting blood tests at the initial visit, triglycerides, cholesterol and blood sugar. There will be feedback on the questionnaires and a discussion concerning lifestyle improvements, if there are any. In the case of abnormal findings in blood samples, blood pressure or extensive alcohol consumption, an appointment will be arranged with the patient’s general practitioner. In cases concerning a need to discuss diet, a meeting will be arranged with a dietician. The patients who are randomised into any of the groups with PAP will also receive a motivational interview and written PAP, which will be based on patient preferences according to the choice of physical activity. There will not be any motivational interview or PAP in the group randomised to only mindfulness,

In this pilot study, the mindfulness groups will include a mix of participants from interventions A and B. In a full-scale study setting there will be separated groups. To increase the number of participants, the courses will be scheduled after normal working hours. The courses will start late in the afternoon and commence as soon as there are 10 participants. The mindfulness course will be led by a trained instructor, and meetings will be held once a week for 8 weeks at the primary health care centre. Participants will also have access to a web-based mindfulness training programme together with an individual login. The web-based training programme can be used with a computer, tablet or mobile phone, regardless of which operating system is used. The web-based course includes a total of 21 h and 36 min of training in eight different levels. The participants will be urged to practise mindfulness with the web-based training programme on a daily basis for approximately 20 min at a time, except on the day of the group meeting. The adherence to the web-based training programme will be measured by minutes spent practising on the web-based programme and with advancement in level.

Three months after the first visit, all the patients will be invited by letter to return for a new set of blood samples and measurements with accelerometer, BMI, blood pressure and surveys. If they do not appear, they will receive a phone call to schedule an appointment time. The patients who received PAP at the first visit will get another prescription in this session with motivational interviewing and written instructions.

After another 3 months (6 months from the commencement of the study), the same measurements, questionnaires and blood samples will be performed as for the two prior visits. At this visit, the patients in the group randomised to only mindfulness will get PAP if needed, as this is standard treatment in inactive patients and so far the most effective way to promote physical activity.

### Measures

**Fig. 2:**
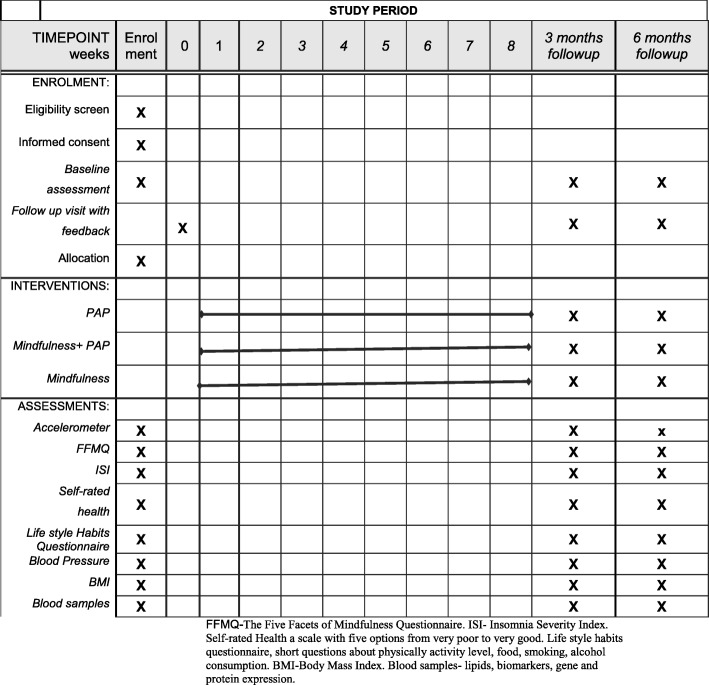
SPIRIT figure: schedule of forms and procedures per study time point

The schedule study procedures along the study time points are presented in Fig. 2.

#### Primary outcome

The primary outcome is the change in minutes of activity, both self-reported and measured by accelerometers (expressed in metabolic equivalent of task (MET) minutes), and compared between the groups.

#### Secondary outcomes

The secondary outcomes are:

1. Changed levels in blood pressure, weight, lipids, biomarkers and gene and protein expression.
2. Changed perceived self-rated health, measured with a five-step scale: very poor, poor, fair, good or very good.
3. Changes in insomnia problem as measured with ISI [23], which assesses the severity of maintaining sleep as well as sleep onset with seven items. The satisfaction with sleep patterns, implication of daily functioning and degree of distress caused by sleep problems are also monitored.
4. Changes in mindfulness measured with FFMQ [22]. This questionnaire consists of 29 statements with the following five facets: non-reactivity to inner experience, observing, acting with awareness, describing, non-judging of experience. The questionnaire uses a 1–5 scale ranging from never/rarely true to very often/always true.

### Feasibility criteria for the pilot study

Several measures for a successful pilot study, i.e. feasibility criteria, will be monitored:

1. Recruitment rate: A recruitment rate of 30% will be considered successful. For comparison, a previous study of exercise training by King et al. had a recruitment rate of only 11% [24].
2. Dropout rate: A dropout rate of maximum 30% will be considered acceptable, which is determined according to the power calculation for the main study.
3. Attendance rate: Another criterion for success is if more than 70% of those randomised to the mindfulness group attend six meetings (75%) or more during the 8-week-long course. The rationale for this cut-off point is that a higher proportion of attendance is assumed to give a higher effect of the training [20].
4. Attendance rate: Another attendance criterion for success is if 70% of patients randomised to mindfulness practice mindfulness for at least 20 min with the web-based application at least 5 days a week [25].

If all the feasibility criteria stated above are fulfilled, the main study can be conducted without further changes in the protocol. If the criteria are not fulfilled, the protocol needs adjustment, and if the criteria are fulfilled to less than 70%, it will not be able to carry on with the main study in its current form.

#### Laboratory values

Study-specific blood samples, drawn after an overnight fast, will be taken at baseline and at both the 3 and 6 months follow-ups. This will be done to analyse eventual changes in lipid profile and plasma glucose. The blood samples will be analysed at the Department of Clinical Chemistry at Helsingborg Hospital. Blood samples will be collected for long-term storage for future analysis of biomarkers, gene and protein expression.

#### Adverse events

Patients will be required to answer questions about adverse advents during the follow-up visits. All side effects will be reported, both positive and negative, and independent of possible relation to the intervention.

#### Statistical and power calculation

This pilot study will be the basis of an upcoming power calculation for a full study. The statistical analyses and results will be delivered approximately 1 year after the study has commenced; i.e. when all the results are sampled from the 90 patients. The number of patients equal to 90 is approximately 10% of a power calculation to a full-scale study.

Preliminary sample size of the full intervention study, with follow-up, is based on a 1:1 relationship between two of the groups and estimated to *N* = 320 in the intervention group (mindfulness + PAP) and *N* = 320 in the control group (PAP only) by group based on a power analysis with 5% significance level and 80% strength. The expected dropout rate is set to 30%. The calculation is based on other studies on compliance to prescribed physical activity outcome measure, where 50% of the subjects followed the recommendation of physical activity by PAP [26]. We expect an increased adherence of 25%, from 50% to 62.5%, in the mindfulness + PAP group at 12 months follow-up.

## Discussion

Physically inactive patients belong to a group who are at great risk of developing different diseases, as reported in previous research [27]. Although many studies have been performed with the aim of finding interventions about getting physically inactive people to become physically active, very few of these studies have been implemented in primary care with sustainability. The implementation of PAP in Sweden is one such intervention that has had success over time. The adherence of about 50% [7] can be explained by the previous data, which showed that it required 12 inactive individuals treated with an intervention like PAP to make one of them increase their physical activity level and maintain it for a year [9]. Thus, it is necessary to try other tools to increase people’s physical activity levels. Patients trust their health care centre to take care of primary prevention, which for most patients includes both screening for diseases and help with behavioural problems such as increased physical activity and smoking cessation [28, 29]. As satisfaction plays a crucial role in behavioural changes, it may make a difference to try to make it easier for patients to feel satisfaction by taking a mindfulness course. In this way, combined with motivational interviewing and PAP, greater number of individuals may be motivated to become physically active. This is especially pertinent when connections have been seen between mindfulness and performing physical activity [14]. Mindfulness is already an accepted treatment in Swedish primary health care, where there now exist mindfulness group sessions with patients who suffer from insomnia, stress, anxiety and depression [20]. Since mindfulness group sessions are an already-accepted treatment, it is easy to broaden their use to inactive individuals regardless of other problems. The combination of physical activity and mindfulness may also provide a greater effect in those with insomnia, depression and anxiety, for which physical activity also has a positive effect.

To conduct the described study, we must be aware of some obstacles. The first is the challenge to engage physically inactive patients to be a part of a study regarding activity. If a person does not have the motivation to take a 30-min brisk walk 5 days per week, we hypothesise that it could be difficult for that person to join a study aiming to increase the activity level significantly. It is possible that the patients who agree to participate are those with a physical activity level just below WHO’s recommendation. It may also be possible that patients will underestimate their physical activity level, with the aim to match the inclusion criteria for the chance to be randomised to one of the mindfulness groups. Even if we try to get the groups to have equal numbers of the same sex, we will likely get the same distribution, with about 60–70% women, as in other studies [30] aiming to increase physical activity levels. We cannot affect this, and we do not know if it is a problem. However, we must take it into consideration when presenting the results. It is also a challenge to include patients quickly enough into the study. Dropouts can increase if it takes a long time between patients getting randomised until the mindfulness group can be started. Since the course is free of charge for the patients and starts in the late afternoon, we hypothesise that this will prevent dropouts. However, the fact that the course is free of charge can also have the opposite effect; i.e. it may not have any value for the patient, who may find it easier to skip group sessions. To evaluate the pilot study, we measure the time spent training with the web-based training programme, but we are also aware that the participants can practise mindfulness without being logged in to the web-based training programme, which can lead to measurement bias. In the concept with PAP there are follow-ups included, which may counteract dropouts. We will also call the patients who do not keep appointments in order to further encourage them to stay in the study.

The reason for using three different groups, instead of two, is to control if only mindfulness itself leads to increased physical activity in this setting. In this way, the study will be a triangulation; the three different treatments will be compared with and tested against each other regarding both physical activity levels and changes in FFMQ to see tendencies—if physical activity increases mindfulness or if it is the other way around. There is a risk, when we have both PAP + mindfulness and only mindfulness in the same group session, that the participants will speak to each other and contribute to an influence to increased physical activity in those who are randomised to only mindfulness. This is an issue to have in mind when analysing the material. However, even if the study participants discuss physical activity with each other, those with only mindfulness will not get counselling based on motivational interviewing or a prescription on physical activity. We therefore believe this limits the increase in physical activity in the mindfulness group due to influence from individuals randomised to PAP+ mindfulness**.** We are aware of the risk, and in a full-scale study there will be, as previously described, separate groups. Moreover, we will be able to compare intervention groups A and B without any carryover effect assessing whether mindfulness in combination with PAP has any additional benefits compared with PAP alone. It can also be a challenge to get participants to return for the follow-ups at 3 and 6 months. Participants who do not appear at the 3 months follow-up after two reminders will be contacted again 6 months from inclusion, if they do not express a will to not participate anymore. The dropout rate is estimated to be approximately 30% in the full study. We hope to have a lower dropout rate and aim to get enough material for an adequate power calculation. We hope to shed some light on the idea that mindfulness interventions can reinforce satisfaction to physical activity and give an increased adherence to PAP in inactive primary health care patients.

### Trial status

The pilot study commenced enrolment on 1 September 2016, and by 21 August 2018 we had recruited 88 participants. The plan is to enrol 90 patients. A total of 54 participants have passed the 6 months follow-up, and there are 12 confirmed dropouts.

## Additional file

Additional file 1: SPIRIT 2013 checklist: recommended items to address in a clinical trial protocol and related documents. (PDF 116 kb)

## Abbreviations

BMI: Body mass index
FFMQ: Five Facet Mindfulness Questionnaire
ISI: Insomnia Severity Index
MBCT: Mindfulness-based cognitive therapy
MBSR: Mindfulness-based stress reduction
MET: Metabolic equivalent of task
PA: Physical activity
PAP: Physical activity on prescription
WHO: World Health Organization
